# First Report of Sorafenib in Patients With Acute Myeloid Leukemia Harboring Non-Canonical *FLT3* Mutations

**DOI:** 10.3389/fonc.2020.01538

**Published:** 2020-08-26

**Authors:** Naval Daver, Allyson Price, Christopher B. Benton, Keyur Patel, Weiguo Zhang, Marina Konopleva, Naveen Pemmaraju, Koichi Takahashi, Michael Andreeff, Gautam Borthakur

**Affiliations:** The Department of Leukemia, MD Anderson Cancer Center, Houston, TX, United States

**Keywords:** sorafenib, *FLT3* mutation, *FLT3-V592*, *FLT3-N676*, AML, TKI

## Abstract

The prognostics implications of patients with acute myeloid leukemia harboring non-canonical *FLT3* is unknown. The use of tyrosine kinase inhibitors in this patient population has not been previously reported. We report successful targeted therapy against non-ITD, non-D835 driver *FLT3* alterations in two patient case studies with acute myeloid leukemia.

## Introduction

The two leading types of *FLT3* mutations found in AML include internal tandem duplications in the juxtamembrane domain (ITD, 17–34%) and mutations in the tyrosine kinase domain (TKD) activation loop (~7%) (1). Mechanistically, *FLT3-ITDs* and *FLT3-TKDs* induce activation of transduction intermediates, including STAT5, AKT, and ERK1/2 (2). Several small-molecule tyrosine kinase inhibitors (TKIs) act as direct inhibitors of FLT3 via competitive inhibition of ATP-binding sites in the FLT3 receptor KD. These include midostaurin, gilteritinib, quizartinib, sorafenib, lestaurtinib, and crenolanib (3–6). Currently, midostaurin (7) and gilteritinib (8) are USA FDA-approved that have activity in *FLT3-ITD* and *-TKD* mutated patients.

However, the prognostic implications and response to TKI therapy of *FLT3* sequence alterations outside the known *FLT3-ITD* and *FLT3-TKD* remain unknown. Frohling et al. identified non-canonical gain-of-function driver mutations (*FLT3 S451F, Y572, V592G, and R834Q*) and bystander passenger alleles (*T167A, V194M, Y364H, and G831E*) in 222 AML patients without a *FLT3-ITD* or *-D835* mutation (9). Functionally, AML cells harboring these driver mutations were amenable to TKI therapy. The overall prevalence of non-synonymous driver mutations in was 1.7% (6 of 349 analyzed samples). Herein, we discuss two patients with AML who acquired non-canonical *FLT3* activating alleles *FLT3-V592G* and *FLT3-N676K*, respectively at relapse and responded to therapy with sorafenib.

## Clinical Summary

### Patient #1

A 66-year-old man was diagnosed with diploid, *NPM1*-mutated, *FLT3* wild-type, *CEBPA* wild-type AML in June 2013. The patient was induced with a clofarabine-based regimen. He was refractory and was re-induced with clofarabine. A Day 21 aspirate revealed 6% blasts. He began therapy with 5-azacytidine administered Days 1–7 every 4–5 weeks and achieved transfusion independence. After cycle 7 a repeat aspirate revealed relapsed AML with 20% blasts. The patient was referred to our institution.

The initial aspirate revealed 24% blasts. A next generation sequencing (NGS)-based analysis for the detection of somatic mutations in the coding sequences of 28 genes (including *FLT3*) was performed on DNA from the bone marrow sample. The methodology of our mutation analysis panel and coverage by genes and codons has been previously published (10) (Supplementary Table 1). The AML was found to have (1) somatic mutations: *NPM1* (W288fs^*^12) and *TET2* exon 3 duplication (T556fs^*^11); (2) variants of uncertain significance: *DNMT3A* exon 17 insertion (V657_D658insE); and (3) a variant of probable germline origin: *GATA2* (P161A). Details for these detected variants are provided in the Supplementary Information. PCR-based DNA analysis did not detect ITD or codon 835/836 point mutations in the *FLT3* (lower limit of detection ~1% of mutant DNA).

The patient received salvage therapy on protocol with E7070, a synthetic sulfonamide cell cycle inhibitor, in combination with idarubicin and cytarabine (ClinicalTrials.gov Identifier: NCT01692197) in June 2014 and achieved a complete remission (CR) with no evidence of minimal-residual disease (MRD) on a multiparametric 19-color flow-cytometry. He received one consolidation cycle and proceeded to allogeneic stem cell transplant from a 10/10 matched unrelated donor in September 2014. The patient achieved 100% donor chimerism by day 28. In February 2015, ~140 days post-transplant, a repeat bone marrow aspirate revealed 66% blasts. The 28-gene NGS panel revealed (1) somatic mutations: in addition to the previously noted *NPM1* and *TET2* mutations the patient demonstrated a FLT3-exon 14 missense mutation (V592G); (2) variants of uncertain significance: in addition to the previously noted *DNMT3A alteration*, an *EZH2* exon 19 mutation was noted (E726Q); and (3) variants of germline origin: including *EZH2, ABL1, IDH1, NOTCH1, TET2* (x3 variants). FLT-ITD and -D835 mutations were not detected by PCR-based DNA analysis. Based on the rapid rate of blast increase, the patient's relapsed disease was more proliferative than the pre-transplant AML.

**Figure 1:**
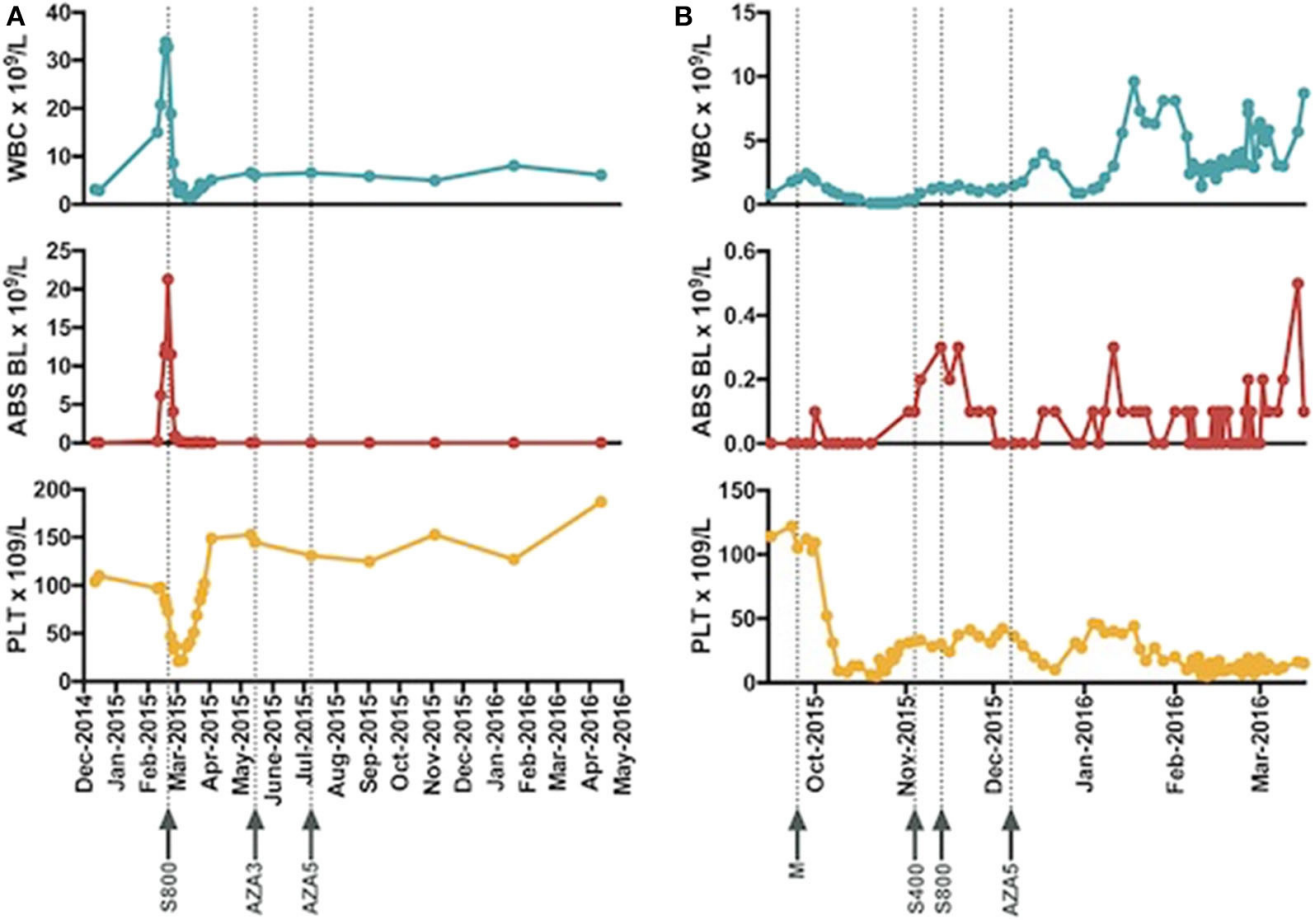
The trend of white blood cell count, absolute peripheral blasts, and platelet and timelines of introduction of sorafenib for patient 1 **(A)** and patient 2 **(B)** are presented. Abbreviations: WBC, white blood cell, ABS BL, absolute blasts, PLT, platelets, S800 (sorafenib 800 mg daily), AZA 3 (azacitidine 75 mg/m^2^ Day 1–3 each cycle), AZA 5 (azacitidine 75 mg/m^2^ Day 1–3 each cycle), M, mutational profiling, S400 (sorafenib 400 mg daily).

**Figure 2:**
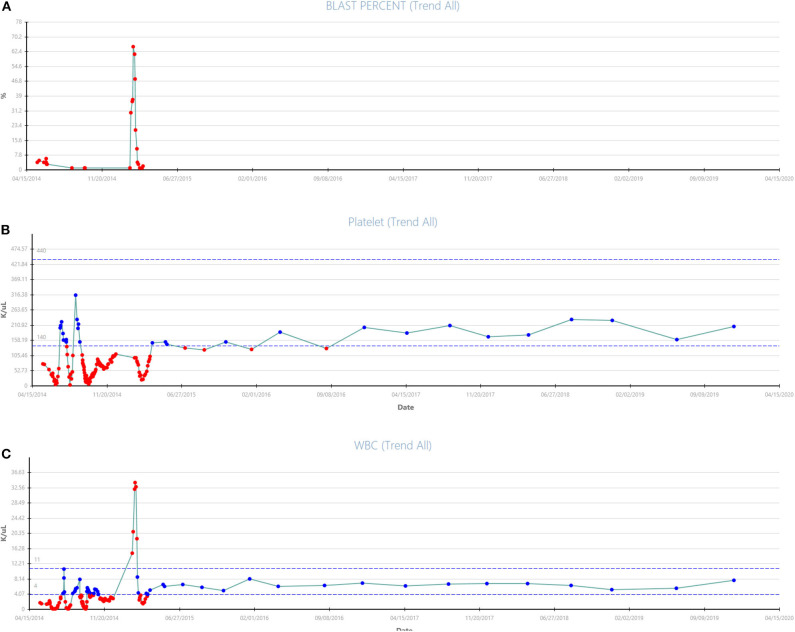
**(A–C)** Updated trend of blast percentage, white blood count, and platelets from presentation in March 2015 to December 2019 with continuation of treatment with Vidaza + Sorafenib combination therapy for patient #1 are shown.

The acquisition of the non-canonical driver *FLT3-V592G* mutation, known to be sensitive to FLT3-inhibition in *in vitro* (9) and known to reside near the juxtamembrane domain, prompted therapy with the multikinase inhibitor sorafenib. The patient started hydroxyurea with sorafenib orally daily continuously at 400 mg twice daily. Three weeks after initiation of the sorafenib the leukocytosis abated and the hydroxyurea was discontinued, in March 2015. An aspirate done 29 days after initiation of the sorafenib revealed CR with no MRD on a multiparametric flow-cytometry. The trend of peripheral blast percentage, white blood cell count, and platelets during sorafenib therapy is shown in Figure 1A. After 3 months of sustained remission with sorafenib monotherapy, 5-azacitidine (75 mg/m^2^ Days 1–5 repeated every 4–5 weeks) was added with the intent to enhance the durability of remission. At the time of this report, the patient remains on sorafenib (200 mg PO BID continuous) and azacitidine (75 mg/m^2^ Days 1–3 every) therapy with the cycles repeated every 4–6 weeks based on count while maintaining MRD negative CR for over 60 months (see updated counts trend in Figures 2A–C).

### Patient #2

A 68-year-old male with a history of myelodysplastic syndrome with diploid karyotype was on observation for 2 years. A surveillance bone marrow aspirate showed 8% blasts with a trisomy 16 in 1 metaphase and diploid karyotype in 19 metaphases. He received four cycles of 5-azacitidine without response and progressed to AML with 20% blast. He received decitabine for 10 days without response followed by fludarabine, cytarabine, G-CSF (FLAG) therapy without response. The patient was referred to our institution in September 2015.

The initial bone marrow at our institution showed 40% blasts and trisomy 16 in eight metaphases. The 28-gene NGS mutation panel identified (1) somatic mutations: *ASXL1* (G629fs), *NRAS* (G12A), *KRAS* (G12D), *FLT3 (*N676K in exon 16), and *GATA2* (M388fs); (2) variants of uncertain origin: *TP53* (M44V); and (3) variants of probable germline origin: *TET2* (P363L). *FLT3-ITD* and *-D835* mutations were not detected by PCR.

The patient received salvage with oral MDM2 inhibitor, DS-3032B, (ClinicalTrials.gov Identifier: NCT02319369) and did not respond. The acquisition of the non-canonical driver *FLT3-N676K* mutation with demonstrated *in vitro* sensitivity to TKI therapy (9) prompted the use of oral TKI sorafenib 200 mg twice daily continuously in November 2015. Ten days into therapy the dose was escalated to sorafenib 400 mg twice daily. An aspirate on day 28 revealed 3% blasts in a hemodilute sample with flow cytometry demonstrating 7.9% aberrant blasts. For subsequent cycles 5-azacitidine was added to the sorafenib in December 2015. He completed 4 cycles of the combination and the repeat bone marrow from March 2016 demonstrated 6% blasts in a hypercellular marrow. Changes in blood counts with therapy are shown in Figure 1B.

## Discussion

In this report of 2 cases we discuss the potential for FLT3-inhibitor based targeted therapy against non-ITD, non-D835 driver *FLT3* alterations in patients with AML. Codon 592 (p.V592) is a part of juxtamembrane (JM) switch motif responsible for maintenance of auto-inhibitory confirmation of *FLT3* in the absence of a ligand and contains a STAT5-binding motif. *V592* is a driver mutation that promotes growth and survival of AML cells by activation of downstream signal transduction cascades in MONO-MAC-6 cell lines (11). Down regulation of *FLT3* by lentiviral transduction of shRNA targeting the FLT3 transcript decreased FLT3 mRNA levels and downstream STAT5 phosphorylation with reduced cell viability (11). *In vitro* studies suggest that *V592G* harboring BaF3 cells are highly sensitive to the FLT3-inhibitor midostaurin (12). The sensitivity of V592G to sorafenib *in vitro* and in patients with AML remained unknown. Our patient with *FLT3-V592G* AML had a rapid response to single agent sorafenib.

The *N676* mutation was discovered in the ATP-binding region of the TKD of the FLT3 gene (13). *FLT3-N676K* is predicted to interfere with FLT3 auto-inhibition by reducing the stability of JM domain and induces phosphorylation of FLT3 and downstream pathways. Huang et al. noted that retroviral expression of N676K induced AML in syngeneic mice with a transforming potential similar to *FLT3-ITD*. Leukemic cells harboring the *FLT3-N676K* mutation in the absence of an ITD mutation were highly sensitive to FLT3 inhibitors quizartinib and crenolanib (14). Our patient with a non-canonical driver *FLT3-N676K* responded well to therapy with sorafenib.

One of the limitations of our manuscript is the lack of *ex vivo* data showing a direct suppression of FLT3 and downstream pathways in these patients by sorafenib. While it may be postulated given the known preclinical activity of sorafenib to FLT3 mutated cells and rapid nature of the responses that the mechanism of action in these patients was likely mediated by the FLT3 inhibitory activity of sorafenib, it is feasible that some of the clinical activity could be driven by multikinase inhibition of prosurvival pathways by sorafenib. This would be an interesting area of investigation for future analysis in non-canonical FLT3 mutations.

Sorafenib is an orally active multi-kinase inhibitor that is 1,000-fold more active against ITD mutant *FLT3* than wild-type *FLT3* in cell based assays (15). Sorafenib has been used for FLT3-inhibition as a single-agent and in combination with other antileukemic drugs in patients with FLT3-ITD AML. Sorafenib is also effective *in vitro* against other ATP-binding mutations including *N676D* and *G697R*. Commercial availability and a broad spectrum of activity against numerous driver *FLT3* allele sequences made sorafenib a logical choice for clinical use in our patients with the non-synonymous driver *FLT3* allele sequences. It is important to note that sorafenib and other type II FLT3-inhibitors have demonstrated no preclinical or clinical activity in FLT3-TKD mutated AML (including *FLT3-D835*) and is generally not recommended to target FLT3-TKD mutant AML. FLT3-TKD mutations are more effectively targeted with type I FLT3-inhibitors such as gilteritinib, midostaurin, and crenolanib.

Routine NGS sequencing of all *FLT3* coding exons will lead to more frequent identification of non-synonymous *FLT3* alleles. After removing known germline variants based on publically available SNP databases we identified 21 patients with non-synonymous *FLT3* allele sequences at our institution since implementation of the 28-gene NGS panel between July 2013 through July 2015 (unpublished data). These include 10 in newly diagnosed untreated AML among 495 evaluated patients (prevalence in new AML = 2%), nine in patients with relapsed AML, 1 in a patient with chronic myelomonocytic leukemia, and one in a patient with myelodysplastic syndrome/myeloproliferative disease. The 21 mutations identified included 12 allele sequences known to have a driver function based on *in vitro* functional studies including *S451F* (*n* = 2), *Y572C* (*n* = 2), *V592G* (*n* = 2), *Y842C* (*n* = 2), *N841K* (*n* = 2), *N676K* (*n* = 1), and *F691L* (*n* = 1) (9, 13, 16, 17). None of these other patients received FLT3 inhibitor therapy. Our experience with sorafenib in the patient with *V592G* and *N676K* suggests that patients harboring known activating non-synonymous FLT3-mutations may benefit from TKI therapy either as a single-agent or in combination with other anti-leukemic agents.

## Ethics Statement

Written informed consent was obtained from the individual(s)/legal guardian/next of kin for the publication of any potentially identifiable images or data included in this article.

## Author Contributions

ND, AP, CB, KP, NP, WZ, MA, MK, KT, and GB collected and reviewed the data, treated the patients, performed the experiments, and wrote the paper. All authors participated in the discussion, have reviewed and approved the current version of the manuscript.

## Conflict of Interest

The authors declare that the research was conducted in the absence of any commercial or financial relationships that could be construed as a potential conflict of interest.
