# Supplementary material for: First Report of Sorafenib in Patients With Acute Myeloid Leukemia Harboring Non-Canonical FLT3 Mutations
Source: Front Oncol. 2020 Aug 26;10:1538. doi: 10.3389/fonc.2020.01538 (PMC7479234; doi:10.3389/fonc.2020.01538)
Supplement: Supplementary file 1 [file Table_1.docx]

**Supplementary Table 1:** Coverage by genes and codons tested for adequate amplicons

**Gene Exons (codons) tested**

*ABL1* (NM_005157) 1-3 (1-99), 3-4 (92-200), 4-6 (195-317), 6-10 (307-523), 11 (560-576), 11 (567-606), 11 (598-638), 11 (661-700), 11 (691-731), 11 (723-762), 11 (752-790), 11 (781-820), 11 (810-851), 11 (843-882), 11 (873-912), 11 (935-975), 11 (965-1003), 11 (995-1034), 11 (1070-1131)

*ASXL1* (NM_015338) 2-4 (20-104), 4-8 (96-253), 8-11 (250-377), 11 (368-407), 11 (398-437), 11-12 (427-589), 12 (581-619), 12 (640-678), 12 (670-709), 12 (701-740), 12 (732-771), 12 (762-801), 12 (792-831), 12 (821-860), 12 (851-889), 12 (880-919), 12 (911-949), 12 (940-979), 12 (970-1009), 12 (999-1038), 12 (1031-1069), 12 (1061-1100), 12 (1091-1129), 12 (1123-1160), 12 (1153-1191), 12 (1182-1221), 12 (1212-1251), 12 (1241-1281), 12 (1271-1311), 12 (1301-1341), 12 (1336-1375), 12 (1369-1542)

*BRAF* (NM_004333) 2-3 (47-157), 3-17 (147-709), 18 (729-767)

*DNMT3A* (NM_022552) 3-6 (25-196), 7-8 (214-322), 9-12 (339-486), 12 (477-492), 14-16 (519-641), 16-19 (631-739), 19 (729-768), 19-20 (759-803), 22-23 (827-913)

*EGFR* (NM_005228) 2-3 (30-96), 3-6 (89-249), 7-9 (273-353), 9-12 (343-444), 12-28 (438-1104), 28 (1096-1133), 28 (1171-1211)

*EZH2* (NM_004456) 2-10 (1-404), 10-11 (395-466), 11-15 (460-610), 15-19 (606-728), 19-20 (719-752)

*FLT3* (NM_004119) 2-3 (15-112), 3-8 (103-311), 8 (301-340), 8-14 (331-610), 14-15 (606-642), 15-24 (635-994)

*GATA1* (NM_002049) 2 (1-16), 2-3 (13-89), 3 (81-120), 3 (111-151), 3 (142-182), 4 (200-248), 5-6 (265-306), 6 (297-336), 6 (377-414)

*GATA2* (NM_032638) 2-3 (1-140), 3 (181-220), 3 (212-251), 3 (242-280), 3 (273-291), 4-6 (318-439), 6 (434-474), 6 (466-481)

*HRAS* (NM_005343) 2-4 (18-129)

*IDH1* (NM_005896) 3-4 (1-100), 4 (92-131), 4-6 (121-228), 6-10 (223-415)

*IDH2* (NM_002168) 1-2 (1-69), 3-5 (106-220), 5-7 (212-281), 7 (278-317), 7 (308-323), 9-10 (361-417), 10-11 (408-453)

*IKZF2* (NM_016260) 2-4 (1-99), 4 (91-129), 4-8 (121-432), 8 (424-463), 8 (454-494), 8 (484-524), 8 (514-527)

*JAK2* (NM_004972) 3-7 (11-220), 7-9 (241-364), 9-11 (359-456), 11-17 (451-723), 17-21 (747-949), 21-25 (939-1133)

*KIT* (NM_000222) 1-3 (1-128), 3 (119-157), 3-5 (152-292), 5 (253-254), 5-6 (292-321), 6-9 (318-460), 9-10 (454-532), 10-12 (523-606), 12-16 (597-765), 17-18 (788-842), 18-21 (834-977)

*KRAS* (NM_004985) 2-3 (1-87), 3-5 (81-189)

*MDM2* (NM_002392) 1-6 (1-134), 7-11 (143-323), 11 (313-352), 11 (342-379), 11 (370-408), 11 (403-498)

*MLL* (NM_005933) 2-3 (145-182), 3 (178-215), 3 (206-244), 3 (236-274), 3 (265-303), 3 (293-332), 3 (325-362), 3 (353-392), 3 (390-427), 3 (418-456), 3 (476-515), 3 (506-544), 3 (534-573), 3 (564-603), 3 (594-632), 3 (624-662), 3 (653-691), 3 (682-722), 3 (714-753), 3 (743-780), 3 (771-809), 3 (799-837), 3-5 (831-1129), 5-6 (1121-1212), 7-12 (1218-1507), 13-15 (1526-1617), 15-19 (1610-1805), 19-22 (1796-1943), 22 (1940-1979), 23 (1985-2024), 24-26 (2032-2114), 26-27 (2111-2186), 27 (2176-2214), 27 (2204-2244), 27 (2236-2274), 27 (2264-2303), 27 (2295-2334), 27 (2324-2362), 27 (2354-2392), 27 (2382-2422), 27 (2413-2451), 27 (2443-2480), 27 (2471-2511), 27 (2502-2542), 27 (2533-2570), 27 (2561-2600), 27 (2591-2629), 27 (2619-2660), 27 (2651-2690), 27 (2710-2747), 27 (2798-2836), 27 (2827-2864), 27 (2856-2894), 27 (2885-2924), 27 (2914-2953), 27 (2946-2983), 27 (2974-3012), 27 (3003-3041), 27 (3032-3072), 27 (3063-3102), 27 (3092-3131), 27 (3123-3161), 27 (3152-3190), 27-32 (3183-3729), 32 (3720-3758), 32-36 (3755-3891), 36 (3882-3921), 36 (3918-3970)

*MPL* (NM_005373) 1 (1-17), 1-3 (7-88), 3 (81-122), 4-7 (146-346), 7 (336-375), 7-9 (370-490), 11 (522-551), 12 (559-636)

*MYD88* (NM_002468) 1 (10-50), 1-3 (41-184), 3 (174-213), 3-5 (211-310)

*NOTCH1* (NM_017617) 1-3 (1-128), 4 (197-237), 5 (248-289), 6 (318-357), 6 (350-367), 8 (419-443), 8 (434-475), 8-9 (466-504), 10 (519-557), 11-12 (632-672), 13-14 (716-785), 16-17 (823-885), 18 (914-981), 20 (1058-1067), 21 (1109-1166), 21 (1157-1170), 23 (1215-1294), 24 (1335-1338), 25 (1373-1413), 25 (1449-1491), 25 (1481-1523), 25-26 (1513-1631), 26 (1625-1666), 26 (1657-1673), 27 (1701-1723), 29 (1795-1804), 30 (1825-1843), 30 (1835-1874), 30 (1865-1880), 31 (1895-1935), 31 (1965-1978), 32 (2012-2028), 34 (2061-2085), 34 (2131-2210), 34 (2251-2293), 34 (2326-2364), 34 (2356-2398), 34 (2420-2460), 34 (2451-2489), 34 (2481-2521), 34 (2511-2549), 34 (2540-2556)

*NPM1* (NM_002520) 1-5 (1-131), 5-11 (123-295)

*NRAS* (NM_002524) 2 (1-37), 3-5 (77-190)

*PTPN11* (NM_002834) 2-3 (5-60), 3-8 (54-299), 9-15 (312-594)

*RUNX1* (NM_001754) 3-4 (20-101), 5-6 (118-205), 8-9 (269-435), 9 (428-465), 9 (460-481)

*TET2* (NM_001127208) 3 (1-22), 3 (13-52), 3 (44-82), 3 (73-112), 3 (103-140), 3 (132-171), 3 (162-200), 3 (191-229), 3 (220-259), 3 (250-288), 3 (279-318), 3 (308-347), 3 (337-376), 3 (369-408), 3 (399-439), 3 (429-468), 3 (459-498), 3 (488-527), 3 (518-557), 3 (549-586), 3 (577-616), 3 (636-674), 3 (665-704), 3 (694-732), 3 (723-762), 3 (752-790), 3-6 (783-1216), 6-10 (1207-1408), 10 (1399-1438), 10-11 (1432-1533), 11 (1523-1563), 11 (1555-1593), 11 (1585-1622), 11 (1612-1651), 11 (1643-1683), 11 (1674-1712), 11 (1703-1740), 11 (1736-2003)

*TP53* (NM_000546) 4 (41-80), 4 (72-112), 4-6 (107-214), 6 (210-224), 7-10 (234-367)

*WT1* (NM_024426) 1 (25-63), 1 (126-204), 1 (197-216), 2 (240-257), 4-10 (291-518)

*Sequencing coverage of the genes: The above table describes adequacy of coverage in our assay across the full set of covered genes, exons, and codons. Adequately covered amplicons are defined as those having total coverage depth of greater than or equal to 250 reads, or for which an orthogonal mutation analysis testing has been performed. Presence of mutations outside the tested regions listed below cannot be ruled out. Due to space limitations, only certain genes may be listed. A full list of covered genes & codons for the specific test results on this sample is available upon request.
